# Diseases of the Antrum

**Published:** 1872-08

**Authors:** E. H. Kilbourne


### ARTICLE II.

### *Diseases of the Antrum.*

BY DR. E. H. KILBOURNE.

Read before the Illinois State Dental Society.

There is perhaps no one of the diseases to which the antrum maxillare is subject, of more frequent occurrence, or that has its genesis more satisfactorily assigned than the one which I propose to review in this brief paper, viz: Abscess. And in approaching the consideration of this subject, it is not to be supposed that I shall bring forward anything particularly new or novel bearing upon the causes and origin of this too frequent affection; for these, to my mind, are already very satisfactorily determined. Neither will I enter upon any discussion of that most obscure of all pathological occurrences—inflammation—believing with Dr. Hartshorn, that while diverse opinions exist as to the essential nature and origin of this process, there is a general agreement among pathologists and clinical observers that its products are substantially the same. I feel no little embarrassment, however, in being met at the threshold with the question, What is abscess, and whether any such affection of the antrum really exists?

This question, so interesting in every point of view, has not been answered alike by all writers. It would appear by reason of a general belief, founded upon the teachings of Prof. Harris and others, that an abscess (properly so-called,) must be a tumor or swelling containing pus, having a homogenous envelope, and never the product of a secretory membrane.

Inflammation, therefore, of the maxillary sinus, terminating in suppuration and abscess, akin to that of the soft parts, must, according to this definition, be among the rarest of all inflammatory events. I cannot believe, however, that such limitation is just, and shall not use the term in any such restricted sense, its derivatives, in my opinion, admitting of a much wider and more liberal range of application, and I can see no good reason for pronouncing a collection of pus seated in the soft tissues, an abscess, and denying the same definition within this chamber, both being products of the same inflammatory action, evolving the same symptoms, and alike also in their destructive tendencies. If a thorn pierce the flesh and remain there, inflammation and abscess ensues.

In necrosis of bone, the meaning of inflammation and suppuration is the loosening and detachment of the dead bone. In like manner, when this chamber is invaded, some more or less pronounced form of inflammation, some unusual cell production is observed, and the intent of the natural efforts, plain and evident. Mucus, a certain portion of which constantly moistens the surface of this, as of every other mucous membrane in health, is altered both in character and in amount by inflammation; and it would be to ignore the revelations of the microscope did we hesitate to affirm that no distinction can be shown between the cells of laudable pus—those loosening and fitting for discharge—a foreign body, a slough, or dead bone, and the changed and altered secretions of this cavity, in a state of chronic inflammation, such as I have been permitted to observe.

Granulations and pus are two forms of cell growth—inflammation, abscess, and suppuration, are simply words applied to the attendant phenomena. There can be no essential impropriety then in classing pathologically all local phlegmasiæ terminating in suppuration, together, as abscesses, their subdivisions being clinical or surgical. I wish then to be understood, that in the employment of the term in this paper, I am not speaking of that rare affection denominated abscess by some writers, (of which there are two or three recorded cases,) but of that diseased condition of the antrum giving rise to all the symptoms and events of abscess, as evidenced in the purulent effusion, (so-called,) but not distinguishable in its microscopical characters or destructive changes from suppurative inflammation in the soft parts, the products of both being alike imprisoned, and differing only in the density of their encasements. But we need say no more to sustain the correctness of these views, it being more important to inquire, what are the causes of this malady?

Ferguson, the distinguished surgeon, says: Inflammation of the lining membrane of the antrum, beginning in the cavity, as it were, is much more rare than most people think; my impression being, that in many, if not most instances, the wall of the antrum is the first part affected, and uniformly assignable to dental irritation. And most writers are agreed in ascribing it to a morbid condition of the teeth and alveoli; Deschamps asserting that if we accept caries of the teeth and diseases of the dental periosteum, which can be communicated to the pituitary membrane, the causes of this affection are unknown. And this opinion was shared by both Fox and our late distinguished countryman and author, Prof. Harris.

It is not denied, however, that its occasional propagation from disease of the nerves may reasonably be anticipated in view of the homogenity and continuity of its tissues, as allied facts may be observed with reference to the propagation of gonorrhœal inflammation. But such instances being unfrequent, and rarely, if ever, coming to the knowledge of our profession, contribute to form the exceptions, and cannot be considered as controverting the correctness of the general rule. And, indeed, the peculiar situation of this chamber, cut off and shielded from every danger other than traumatic, and the possible introduction of some irritating agent through its natural opening, an occurrence most improbable, lends additional support to these views. It is not known what influence, if any, occupation, climate, or other circumstances exert upon the development of the disease. Just how far, or to what extent also, constitutional vice or habit of body of any kind can be held responsible for the initiation or continuance of the same, I am not in a position to state; but that it is a remote or predisposing cause, and exercises an influence, however small, in determining the form or malignancy which the disease assumes, there can be no manner of doubt. But according to the fullest possible allowance to these causes, I think it may safely be assumed, that in a very large majority of the cases of disease of this chamber, those morbid conditions of the teeth and gums giving rise to irritation in the alveolar periosteal tissue, must be held primarily responsible. It occasionally happens (twice in my own experience) that the apices of the roots of one or more of the teeth immediately subjacent (preternaturally long) penetrate the floor of the antrum, and becoming situated in it, light up an inflammation, which, if not speedily arrested, eventuates in the formation of an abscess.

Prof. Baum relates a case where this cavity was distended with purulent matter in consequence of the lodgement of a tooth in its interior. It is of little practical consequence, however, as it regards the morbid effects produced upon the lining membrane and surrounding bony parietes of the antrum, remarks Dr. Harris, whether the abscess be found in it, if the matter be discharged there, or in the alveolus of the tooth that gave rise to it. The effects are about the same in one case as in the other.

I abstain from giving here any groupings of the symptoms usually attendant upon this affection, for they are not positive nor constant, and only such as to warrant a probable diagnosis. Nevertheless, the information furnished from the history of the case, and the intelligence afforded by the objective symptoms alone are commonly quite sufficient to indicate pretty clearly the nature of the complaint; and as the important and more or less constant symptoms will be enumerated in the history of the two cases which I am about to relate, I shall hasten to the consideration of that which is of greater interest, and perhaps more practical importance. And here, then, I come to one of the chief objects of this paper. And as every pains-taking inquirer, notwithstanding the frequency with which the ground has been turned, seems to make fresh discoveries, I hope it may not be without interest to state briefly the result of my experience, and to call your attention to certain advantages in the treatment of this affection, which, as yet, I believe are not very widely known or practiced.

I will first, however, consider some of the curative indications in the treatment of this disease. And, under this head, I feel that I cannot do better than quote from the writings of Prof. Gross, even at the risk of repeating what has been long known and universally accepted. He says: "The treatment of abscess of the antrum is conducted on the same principles as that of the abscess of the soft parts. The rule is, to afford a free outlet to the pent up fluid, if possible, before the occurrence of serious structural change; the evacuation being effected at the most dependent portion of the antrum. Such a step is not neglected, even when there is no material obstruction in the natural orifice of the insufficiency of this from its elevated, and consequently unfavorable position, being well known." As the disease is generally dependent upon the irritation of a decayed tooth, (the fang of which often projects into the sac of the abscess, and only requires removal to let out its contents,) it should of course be extracted, together with other diseased teeth and stumps of teeth, if any, even when it is not very apparent that they are the cause of the inflammation. Should the opening thus made be inadequate, it can be enlarged and carried into the antrum by means of a trocar or drill, and escape given to the retained matter. Patency is carefully maintained until the mucous membrane has regained its normal functions, an occurrence which may often be greatly expedited by the use of mildly astringent injections and suitable constitutional measures."

In my opinion, however, nothing is to be expected from medicinal means in this disease, any more than in similar affections in other parts, and hardly as much, for in this case no vital tract is concerned, and the departure from health is not commonly so great as it is in most other affections of this membrane. Generous diet is of course called for, and if the systemic disturbance be great it should be corrected. Anything that will assuage pain and quiet the nerves should be employed. Experience favors the use of hot fomentations to the face when the pain is acute.

But by far the most potent weapon in discussing and removing this disease will be found in the local applications recommended above; this being the old conventional treatment for this affection, and which the aggregate testimony of the profession for years has approved and sustained. But how can it the most effectually be done; and, if a choice, what method is preferable? I refer now to such cases as are beyond the power of hygiene or constitutional measures of any kind, and where the lining membrane of the antrum is in a state of sub-acute or chronic inflammation, pouring out a thick purulent fluid, not infrequently fetid in character. In my earlier experience I had met with no little difficulty in treating successfully this class of cases, and principally on account of the opening to this chamber (usually effected through the socket of an offending tooth,) being commonly too small to admit the use of a syringe and the introduction of a stream of sufficient volume and power to thoroughly bathe and cleanse the whole internal surface of the mucous membrane and walls of this cavity. And as I write, there comes up the too vivid recollection of a case where I was baffled and defeated, I have the best reason to believe, because of this very difficulty and defect.

Yet we are told, *ex cathedra* as it were, that the affection is one easy of cure, and that in the immense majority of cases, the progress towards recovery is singularly uniform. On the contrary, the reverse according to my experience, has been remarkably the case; so much so, that I have long been surprised at the statement, and I venture to say, that such views are *contrary* alike to *reason* and *fact;* for all our knowledge on the subject tend to show that it depends on the health and condition of the individual patient, which is usually much depressed. And in coming to these opposite conclusions, either I must have had an unusual complement of bad cases, or these writers a wonderful exemption from them.

I have no wish, however, to depreciate their treatment, or question their success in the employment of the syringe; yet I feel warranted, after a somewhat extensive experience, covering a period of over 25 years, in asserting, confidently, that injections, by the ordinary means now in use, and recommended in the text books, are quite inadequate to the cure of the severer forms of the disease, (at best, always tedious,) and though exceptionally efficient, their general use will bring disappointment and vexatious delay. And this opinion finds corroboration in the experience of many of my brother practitioners.

Deeply impressed, then, with the want or success and annoyance of the practice above described, I experimented with, and have lately come to adopt the method and instrument now in such universal favor for the treatment of catarrh, viz: "Thudicum's nasal douche." Its utilization to the treatment of diseases of the antrum was first suggested to me by witnessing its operation upon a little girl suffering from catarrh, and upon watching its effect and the simplicity of its accomplishment, I became convinced that nothing could rival the excellence of this particular practice in the local treatment of diseases of this chamber. And not long afterward I was afforded an opportunity of putting it to the test, and with such gratifying success as to warrant me to continue its use and to bring it prominently before the profession.

The apparatus, simple in construction, is too well known I think, to require description. It can be improvised in fifteen minutes in the office of any practicing dentist, by attaching a piece of rubber tubing (small size) three or four feet long to the mouth of a quart bottle, previously filled with water, and suspended from a ring above, inverted, or neck down, the free end of the pipe holding a conical button or cylinder of silver or other metal, so shaped that it can be carried into the sinus, through its alveolar opening, and your instrument is complete. Now, this little contrivance, simple, inexpensive, and easily made, is, in my opinion, a desideratum in the treatment of these affections hitherto unknown.

Among the advantages which may be enumerated in favor of this practice may be mentioned: 1st. Its greater safety and ease of application. 2d. The ability of the patient to use it alone at home. 3d. The clearness of the cavity is assured, the fluid reaching and acting upon every portion of the mucous lining. 4th. Duration of treatment notably abridged.

In consequence of a skepticism, for which I trust I shall be pardoned, in regard to alleged extraordinary merits of the thousand and one astringent wishes recommended by different authors for this affection, I have been inclined of late to make trial of the virtues of cold water alone; the therapeutic value of which, in the treatment of many diseases has long been recognized; its tonic and constringing influence being well known to act most happily in promoting the healing of ulcerated surfaces. But the constant and persevering use of it as a remedial agent in the treatment of diseases of the antrum, (like the practice now in vogue for the treatment of catarrh,) has not, in my judgment, heretofore been advocated.

I have not had the leisure to make an exhaustive search of the journals, and may possibly be claiming credit for ideas already advanced by others; but if so, I have no knowledge of them.

Cold water is a harmless and yet effective stimulant to the mucous membranes, possessing value far beyond the vaunted virtues of many of the drugs now in use. And in the absence of a rationale by which its special applications might be definable, I would recommend its employment in all those diseased conditions of the antrum calling for the local influence of detergent remedies. A compromise, I imagine, goes far to settle what may be deemed the best application in these affections, and some, no doubt, will choose to associate carbolic acid with the water, and although I do not mean to deny the occasional necessity for its employment, I am strongly of the opinion, from my own experience, that its general use is not demanded. In order however, to assign a marked effect to any therapeutic agent, it is necessary first to inquire whether it will, with any certainty, effect a cure in cases that have resisted other methods of treatment. I regret that I have not had more abundant opportunities for testing this; but as far as can be judged from the few experiments made with it, I feel justified in concluding, that if used with thoroughness, (made possible only with the douche) it will control and dispel the inflammatory action in this sinus with more certainty and celerity, and with less annoyance to the patient and operator, than any remedy or association of remedies yet proposed.

The cure in some instances is affected with amazing rapidity, after many other medicaments have been applied without effect. A good illustration of what I state has been recently afforded me in the case of a young lady, (Miss Chase,) who had been under treatment at Ann Arbor some six months previous to coming under my care, having been sent there by some brother dentist, whose time and patience had been worn out in the chase after some remedy for this chronic case. On inquiring into her history, I found that some two years previous she was seized with what she denominated neuralgic pains in the right cheek and temple, frequently visiting the teeth upon this side, which for some time had been preternaturally sensitive and sore, the pain persisting with such severity that she was obliged to seek the advice of her physician, who, suspecting the true nature of the complaint, advised her immediate attendance upon a dentist; but having a fear of his services, she thought it "better to stand those ills she had, than fly to others she knew not of," and so delayed the necessary visit until the pain becoming intolerable, she finally consented to the removal of a perfectly sound molar tooth, on this side of the jaw, which gave escape to a large quantity of purulent matter from the antrum, and complete relief from all the painful symptoms.

She at once placed herself under the care of this dentist, who treated her with as much zeal and skill, judging from her account, as was possible to bestow on the case, using medicated injections of all kinds faithfully, for upwards of six months, and yet found himself impotent to arrest the disease.

About this time the lady went upon a visit to some friends in Ann Arbor, having previously obtained the consent of her dentist—only too willingly given, I apprehend, that he might escape the mortification of defeat. And here she sought counsel and treatment from one of the faculty in the medical department of the University, where she remained a long time, submitting to nearly the same course, by injections, *ad infinitum*, associated with constitutional treatment, that she had hitherto undergone at home; but all to no purpose. I must not forget to add, that during all this time she reported her health as being very poor, though remarkably improved since then.

In this condition and with this history, she applied to me, some few months ago, for relief. Certainly not a very cheering picture, or a very promising case. Encouraged, however, by my faith in the potency of the douche, I consented to undertake it, though with no very strong hope of success. Upon examination, the teeth and gums were found measurably healthy, and over the site of the first molar a small opening leading into the antrum, large enough to admit the handle of a small excavator, from which exuded a thin, purulent fluid of more or less constant production. Further explorations with the probe revealed a thickened condition of the lining membrane of this chamber, with its outer wall rough, having the feel of caries. I at once enlarged the opening by means of a sponge tent, and on the following day applied the douche some three or four times, using nothing but cold water, it giving no unpleasant sensations. The application was repeated every day for a week, at the expiration of which time there was a marked improvement in the discharge. Its use was now persevered in for upwards of three months, using nothing but pure cold water, to which was added, at the very last, a few drops of carbolic acid; and the history of the case from this time on, was that of a steady and rapid decline of the affection, resulting in complete restoration and cure, in the short space of between three and four months.

*Case* 2.—Mrs. H.————, married, æt. 40, of a pale, nervous, depressed aspect, health thoroughly out of order, and the tongue foul, presented herself for treatment last October, complaining of deep seated pain in the left cheek, of a throbbing pulsatory character, extending from the alveolar border to the lower part of the orbit, located principally in and above the first molar, and at times, wholly confined to that tooth; the pains being occasionally sharp and lancinating, flitting about over this side of the face with great irregularity, but usually more severe in the morning, coming on about 9 o'clock, and lasting some two hours, when it would intermit, and recur again with great severity, varying from one to twelve hours. The tooth, sensitive on percussion, was comparatively healthy; yet, after a careful diagnosis, I advised its extraction. After some thought, she consented, and on removing it and puncturing the floor of the antrum, a thick purulent discharge of a tablespoonful or more followed the withdrawal of the instrument, which gave immediate relief. The cavity was cleansed with warm water, and the patient, very weak and nervous, was allowed to depart, assuring her that the extraction of a tooth was merely fencing with the outside of this disease, and urging her attendance on the morrow. She complied, when I again made use of the douche, using nothing but cold water, and with the same frequency as in the previous case, the patient using it alone at her home a good portion of the time, and with the same happy results as before; the disease rapidly disappearing, and recovery pronounced in about four months.

In concluding, let me ask, what is the explanation of the unusual success in the treatment of these two cases just reported? For it will not be disputed that the first was exceedingly intractable and rebellious, having a chronicity that was truly appalling; and the second, a fair type of the acute variety more commonly met with, and both illustrative cases. To my mind, it seems clear that these results were not reached, so much by virtue of any unusual care bestowed on the cases during the progress of the treatment, as through the obvious merit and efficiency of the means employed; and while all admit that the old method gives not unfrequently very satisfactory results, yet the very considerable proportion of failures which follow its use, even by the most skillful, leads to the conviction that the adoption of the new one here extolled, cannot be too forcibly pressed upon the dental profession, and I trust that others may think it worth while to make use of this method in this, and kindred diseases of the antrum, and publish the results of their investigations.

